# Transperineal ultrasound as a reliable tool in the assessment of membranous urethra length in radical prostatectomy patients

**DOI:** 10.1038/s41598-021-81397-z

**Published:** 2021-01-19

**Authors:** Kania Piotr, Mieleszko Rafał, Kuligowski Marcin, Dudka Karol, Kuca Monika, Biedrzycki Jakub, Zwolan Bartosz, Dmowski Tadeusz, Salagierski Maciej

**Affiliations:** 1 Department of Urology and Urological Oncology, St. John Paul II Mazovian Provincial Hospital in Siedlce, Siedlce, Poland; 2 Department of Urology, Collegium Medicum, University of Zielona Góra, Zyty 26, 65-046 Zielona Góra, Poland

**Keywords:** Oncology, Urology

## Abstract

To evaluate the usefulness of transperineal ultrasound (TPUS) as a method of membranous urethra length (MUL) measurement and investigate whether preoperative (MULpre) and postoperative (MULpost) would be associated with the degree and time of urinary continence recovery after laparoscopic radical prostatectomy (LRP). 84 patients who underwent LRP between January 2017 and December 2018 were selected for final analysis. All patients had preoperative and postoperative measurement of MUL in TPUS. Urinary continence was defined as no pad or a safety pad. Recovery of continence was assessed at 1, 3, 6 and 12 months after catheter removal. We prospectively analyzed correlation of MULpre, MULpost and a percent change in membranous urethral length (MULratio) with the urinary continence status. 69 (82%) patients regained continence in the follow-up of 12 months. MULpre, MULpost and MULratio assessed in TPUS were larger in subgroups of patients who regained continence earlier and in the entire continent group. Spearman rank test showed strong correlations between MULpost and MULratio (R—0.6 and R—0.56, respectively, p < 0.0001) with the time to continence recovery in the cumulative 12 months follow-up. TPUS allowed a reliable measurement of MUL before and after LRP. MULpre, MULpost as well as MULratio are related with time to regain continence and recovery rate after LRP. Sparing longest possible sphincteric urethra, with respect to oncological outcomes is a key factor in recovering continence after prostate cancer surgery.

## Introduction

Prostate cancer as a one of the most common malignant disease in man in majority of cases is diagnosed in its organ confined and localized stage. Laparoscopic radical prostatectomy (LRP) can be offered to these patients as a definitive treatment option. Although postprostatectomy incontinence (PPI) is the most debilitating complication and significantly impairs the health related quality of life, overwhelming number of patients will regain continence in time. Numerous factors were taken into account in attempts to predict PPI^1^. Efforts in assessing this risk will help patients and urologists in the decision-making process before surgery, provide them with realistic expectations and may lead to the implementation of additional modifications during surgery or intensification of perioperative physiotherapy^2^. Reported outcomes of PPI is not easy to assess and compare as it can be influenced by preoperative patient characteristic, surgeon experience, surgical technique and method used to collect and report data and PPI definition^3^. Preoperative, patient-related factors that can potentially increase the rate of PPI and time to continence recovery after RP include advanced age, higher body mass index (BMI), deficiency of physical activity^4^, large prostate size or intravesical protrudance of prostate into the bladder, comorbidities^5^, former prostate surgery and lower urinary tract symptoms (LUTS). Preoperative membranous urethra length (MULpre) measured in magnetic resonance imaging (MRI) was reported to be a patient-related anatomical factor that can significantly affect continence recovery following RP^6,7^. In the meta-analysis done by Mungovane et al. mean MUL in MRI ranged from 10.4 to 14.5 mm and individual MUL can be as small as 5 mm and/or as large as 34.3 mm^8,9^. As the entire length of membranous part of urethra is covered by active components of sphincteric complex and is called sphincteric urethra, the use of preoperative MUL as an independent predictor appears to be justified. Many studies on the basis of preoperatively measured MUL concluded that sparing of possibly longest sphincteric urethral length during RP is one of the key factors to achieve higher continence rate and earlier continence recovery^7,8^. Unfortunately, there is a scarcity of data on the postoperative MUL. The length of postoperatively measured MUL (MULpost) can have even more significant influence on continence rate since it depicts the length of sphincteric urethra that could have been spared during surgery^7^. Postoperative MUL depends on both MUL before the operation and the quality of the dissection and anastomosis during surgery. In most studies, MUL was measured in MRI as this imaging is commonly used in preoperative setting. Transperineal ultrasound (TPUS) is widely used for measurement of urethra length in women^10^. TPUS was also implemented in examination of male pelvic organs in static and active situations^11,12^.

Our study, of prospectively evaluated cohort of patients who underwent preoperative and postoperative TPUS assessment of MUL, investigated whether these findings are related to PPI ratio and time to continence recovery after LRP.

## Methods

### Study population

The protocol of this study was reviewed and accepted by an Institutional Review Board (decision no 74/2017). We do confirm that all methods were performed in accordance with the relevant guidelines and EU regulations. The study cohort included patients who underwent LRP between 2017 and 2018 by a single experienced laparoscopic urology surgeon (P.K.). Patients presenting with overactive bladder caused by neurological diseases (n = 4), who underwent simultaneous laparoscopic abdominoperineal resection of rectum and LRP (n = 1), with postoperative anastomosis stricture requiring endoscopic treatment (n = 1), patients who received adjuvant irradiation before regaining continence (n = 4) and those with missing complete follow-up data (n = 26) were excluded from the study. A total of 84 patients were eligible for the final analysis.

### Preoperative assessment

**Figure 1:**
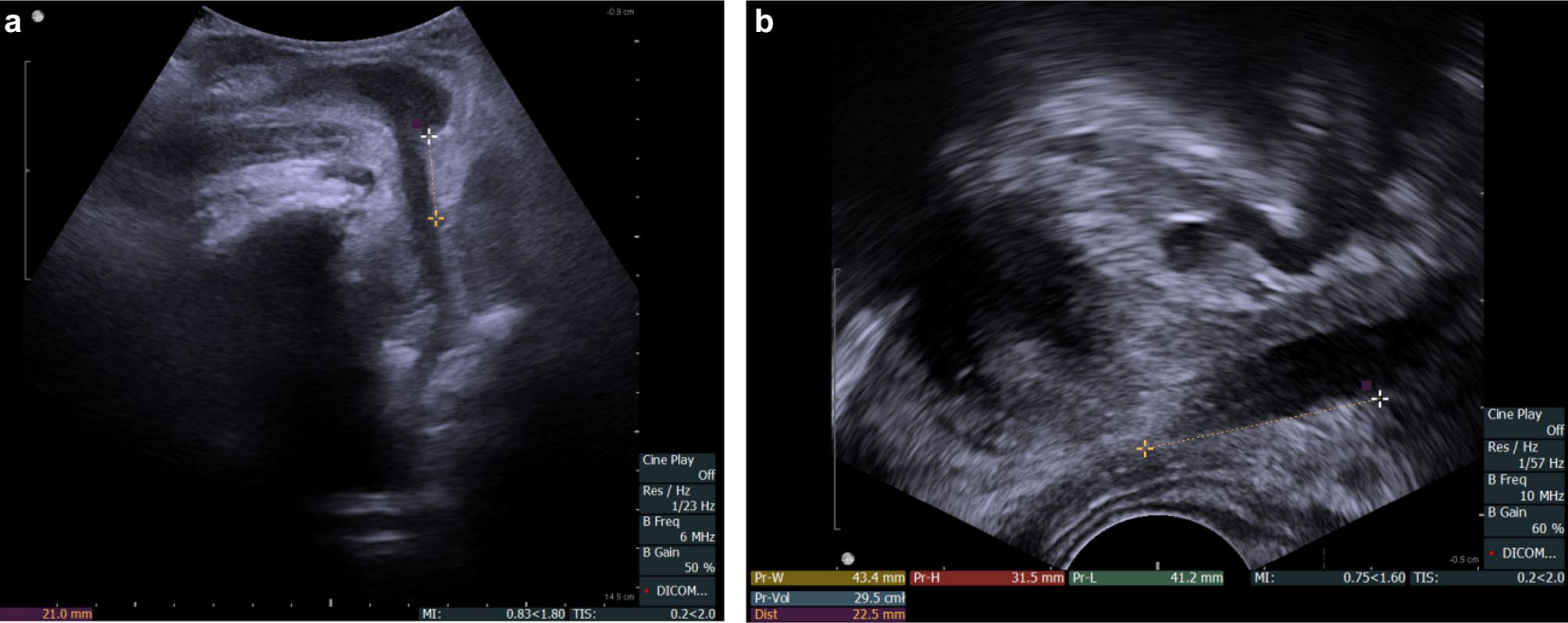
(**a**) Preoperative membranous urethra length—MULpre = 21 mm, measured in transperineal ultrasound—TPUS. (**b**) Preoperative membranous urethra length—MULpre = 22.5 mm, measured in transrectal ultrasound—TRUS.

All patients during routine preoperative evaluation underwent assessment with transrectal (TRUS) and transperineal (TPUS) ultrasonography. The TPUS and TRUS imaging procedures were undertaken using a BK Flex Focus 400 (BK Medical, Denmark) ultrasound machine, standard curved array transducer 8830 (2–6 MHz) and prostate biplane 8808e (5–10 MHz) transducer. In preoperative assessment the patient was asked to consume 300 mL of water 30 min before examination. The examination was performed in supine position with knees flexed to 70–90 degrees and hips slightly rotated outwards. The membranous urethra length (MUL) was defined as a distance from the surface of prostate apex to the entrance of urethra into the penile bulb and was measured along the posterior surface of the urethra. MUL was measured on a midsagital plane although in some cases it was possible to depict the whole length of urethra also in coronal plane that confirmed the urethra length. The mean lenght, out of three measurements, was taken to final analysis. During TPUS, all patients were additionally instructed on the pelvic floor muscles training (PFMT) and could observe anterior movement of membranous urethra on the screen—visual biofeedback. Patients were encouraged to perform PFMT before surgery and after a catheter removal. As a standard preoperative assessment, patients were also examined with transrectal transducer. TRUS was used to measure the prostate volume, depict intravesical prostate protrusion and the shape of prostate apex. It was demonstrated in studies that a considerable part of sphincteric urethra can be covered by the parenchyma of prostatic apex^13^. Sparing of a maximal length of sphincteric urethra during surgery can be individualized on the basis of the data from TRUS or MRI. MUL was also measured in TRUS to compare with TPUS measurement (Fig. 1a,b). At the time of preoperative consultation patient characteristics including age, comorbidities, smoking status, body mass index (BMI) and tumor characteristics—clinical tumor stage (cT), ISUP grade group and preoperative prostate specific antigen (PSA) were collected.

### Treatment

The surgery was carried out through transperitoneal access when extended lymphadenectomy was performed and extraperitoneally in cases were extended lymphadenectomy was not recommended. All the procedures were performed utilizing three-dimension Image1 S 3D endoscope (Karl Storz, Germany). During the procedure special care was taken in apical preparation to achieve longest possible length of membranous urethra with posterior urethral wall dissection just distally to the seminal colliculus. The anastomosis was preceded with a single suture, reapproaching the remnant of the rectourethralis muscle and the medial dorsal raphe with the posterior aspect of the bladder neck. This suture prevented the distal contraction of the urethral stump, provided a posterior support for the anastomosis and enabled a tension-free anastomosis. During the anastomosis, that was performed with running suture, special care was taken not to make too deep needle passage through the urethra, as it could lead to atrophy, scarring and further shortening of the sphincteric urethra.

### Postoperative assessment

**Figure 2:**
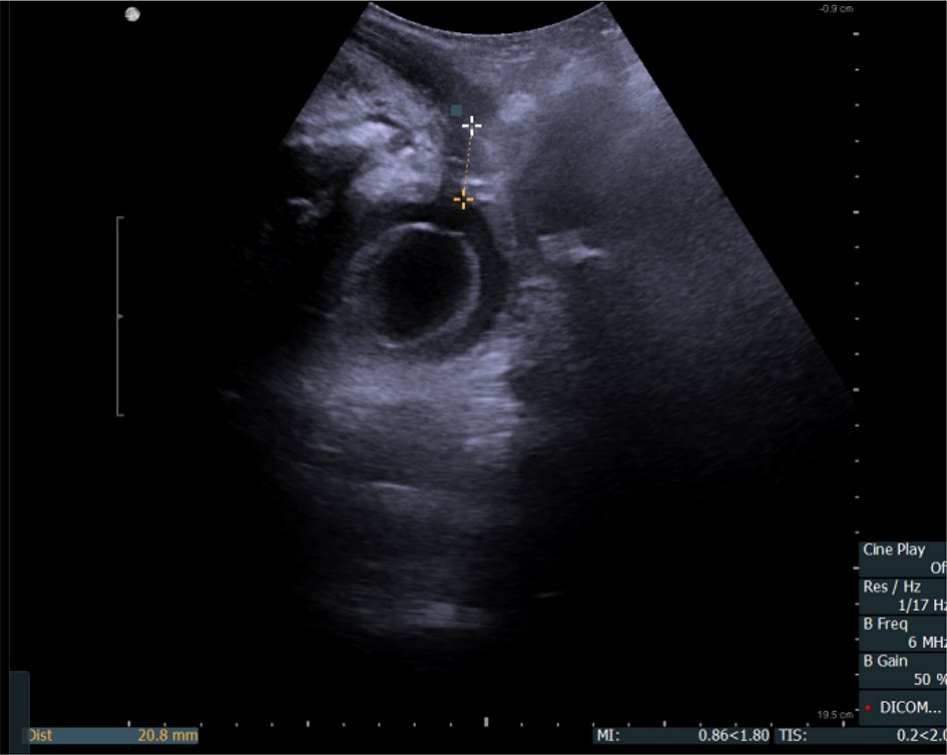
Transperineal ultrasound (TPUS) postoperative membranous urethra length (MULpost) measurement—MULpost = 20.8 mm. MULpost was measured after gentle cranial displacement of the catheter, 8 days after surgery.

**Figure 3:**
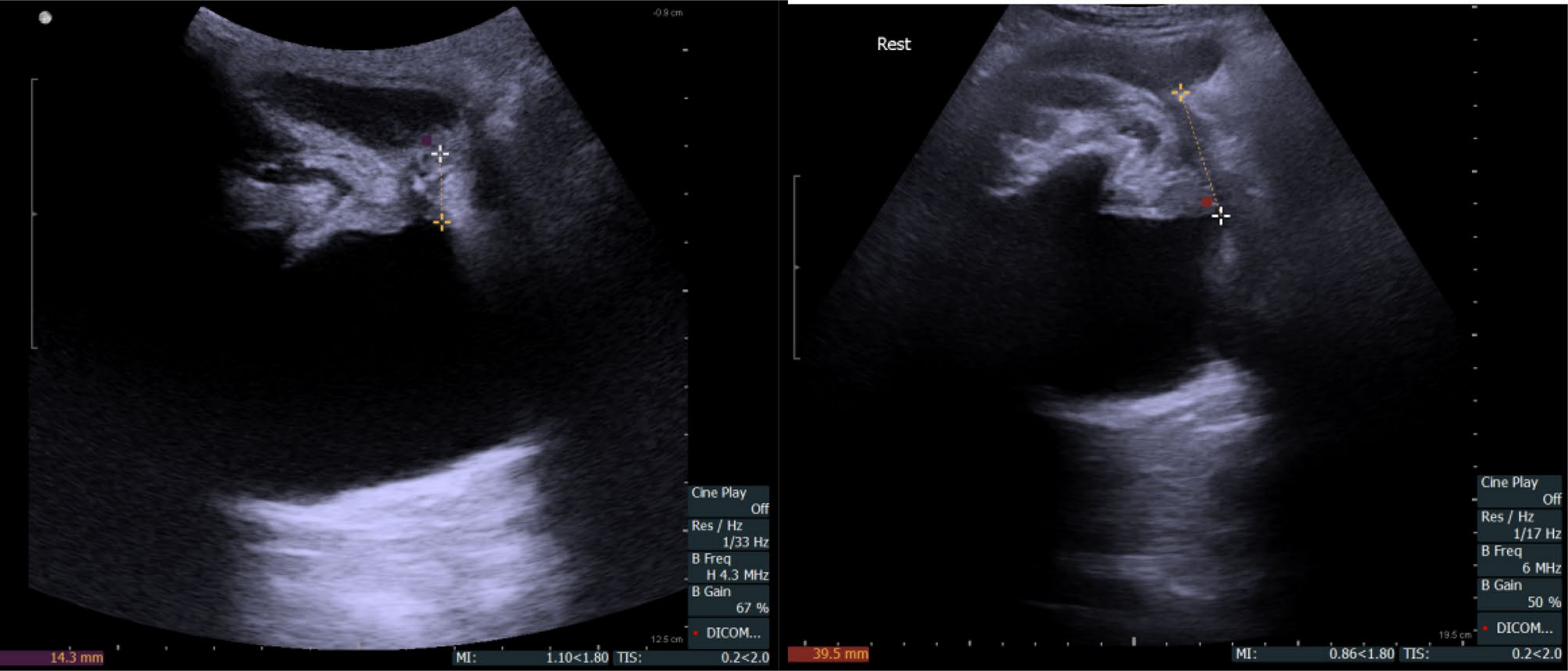
Transperineal ultrasound (TPUS) postoperative membranous urethra length (MULpost) measurement—left side, MULpost = 14.3 mm—patient incontinent at 12 months after surgery, right side, MULpost = 39.5 mm—patient continent at 1 month after surgery.

The first postoperative assessment was performed on day 7–12 (mean 8) days. TPUS examination was performed just before removing the catheter with bladder filled with 100 mL of saline the membranous urethra length (MULcath) was measured from penile bulb to the surface of the bladder with catheter balloon gently pushed cranially (Fig. 2). The final length of membranous urethra (MULpost) was measured with TPUS during the assessment one to three months after catheter removal (Fig. 3). As the difference between MULpost and MULcath was almost negligible, only MULpost was considered appropriate as postoperative MUL for the analysis. The percent MUL change was calculated as [(MUL post − MUL pre) × 100]/MUL pre and presented in the study as a MULratio.

### Outcome assessment

Expanded Prostate Cancer Index Composite (EPIC) survey^14^ was used to evaluate continence on 1, 3, 6 and 12 months after catheter removal. The continence was graded on a 4-graded scale (Table 1). Patients were asked to record the number of pads used per day during postoperative follow-up period. Time to final, stable continence was recorded as number of days after catheter removal to achieve level of continence 0—no pad or S—safety pad.

**Table 1:** Levels of final continence after LRP.

The level of final continence		
0	Complete continence	Continent
S	Safety pad	Continent
1	One pad daily	Incontinent
2	Two or more pads daily	Incontinent

### Statistical analysis

Statistical analysis was performed using Statistica 10.0 software (TIBCO Software, CA, USA).

### Ethics approval and consent to participate

The study was performed with ethical approval (decision no 74/2017, 12th December 2017, received from Bioethics Committee at The Polish Mother’s Memorial Hospital Research Institute) and patients informed verbal and written consent.

## Results

### Patient characteristics and preoperative membranous urethra length

**Figure 4:**
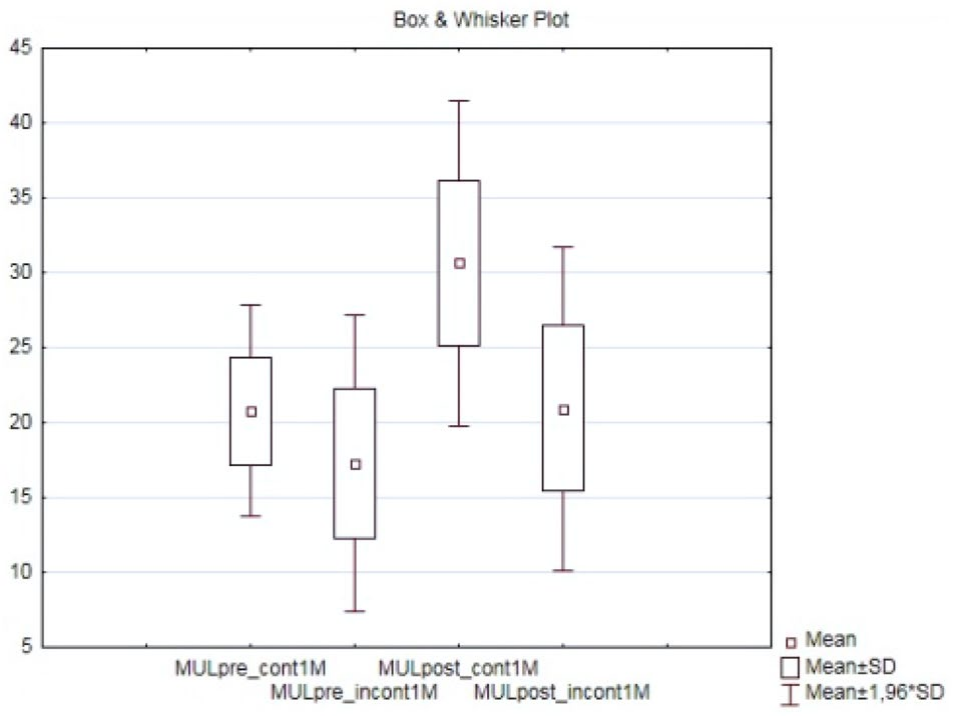
Comparison of membranous urethra length measured preoperatively (MULpre) and postoperatively (MULpost) in a subgroups of patients continent (cont 1 M) and incontinent (incont1M) wihtin one month after catheter removal; (CI 95%, p = 0.0005).

**Figure 5:**
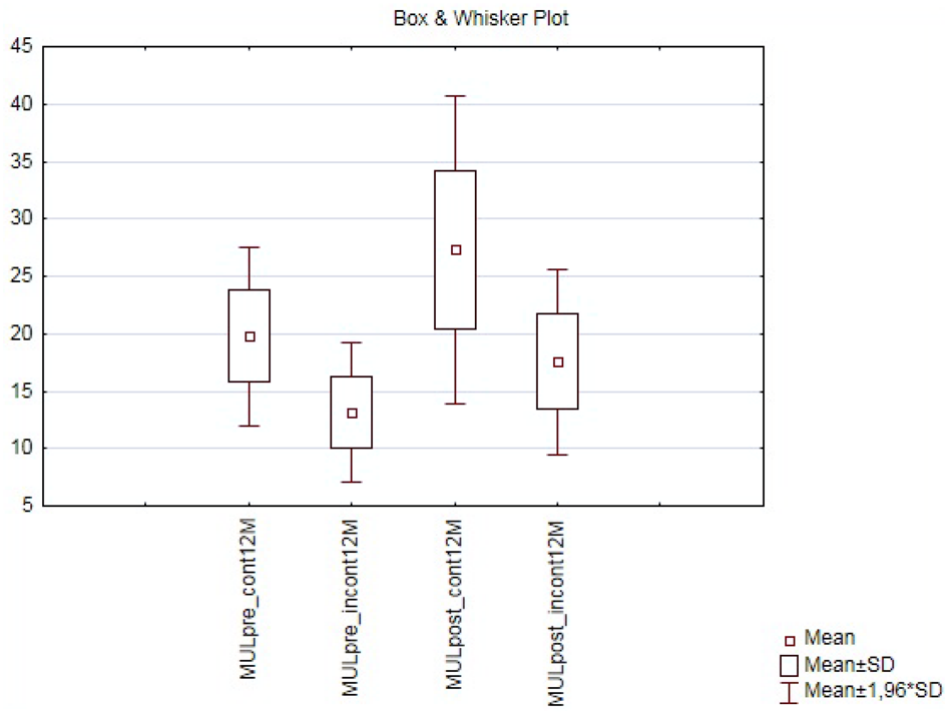
Comparison of membranous urethra length measured preoperatively (MULpre) and postoperatively (MULpost) in a subgroups of patients continent (cont 12 M) and incontinent (incont12M) after 12 months follow up; (CI 95%, p = 0.0005).

Preoperative MUL (MULpre), clinical and pathological characteristics of 84 patients included in the study are presented in Table 2. Median patient age was 66 years (IQR, 61–69). Range of MUL measured preoperatively in TPUS was 10–30 mm, median MUL 20 mm (IQR, 15–23) and was comparable with MUL measured in TRUS 19 mm (IQR, 15–22). Preoperative MUL was significantly longer in a subgroup of patients continent within one month after catheter removal (p = 0.0005) as well as in the entire group of patients continent after 12 months of observation (Figs. 4, 5), although MULpre did not show strong correlation in Spearman’s rank coefficient with time to continence recovery.

**Table 2:** Clinical and demographic characteristics of patients included in the study (n = 84).

Patient characteristic (n = 84)	Mean (± SD)/Median (IQR)
Age (years)	65.2 ± 5.3/66 (61–69)
BMI—body mass index (kg*m^−2^)	28.2 ± 4.2/27 (25–31)
PSA (ng mL^−1^)	9.2 ± 5.8/7 (6–11)
Prostate volume (mL)	34.4 ± 15.2/(25–39)
Time of surgery—mean (range)	180 (90–264)
ISUP grade at biopsy	n (%)
1	44 (52.4)
2	28 (33.3)
3	7 (8.3)
4	4 (4.8)
5	1 (1.2)
Clinical T stage	n (%)
cT1	50 (59.5)
cT2	32 (38.1)
cT3	2 (2.4)
Preoperative MUL	Mean (range)/Median (IQR)
measured in TPUS (mm)	18.9 (10–30)/20 (15–23)
measured in TRUS (mm)	18.5 (10–31)/19 (15–22)

*BMI* body mass index, *PSA* prostate specific antygen, *ISUP* International Society of Urological Pathology, TPUS = transperitoneal ultrasonography, *MUL* membranous urethra length, *TRUS* transrectal ultrasonography.

### Postoperative membranous urethra length

**Figure 6:**
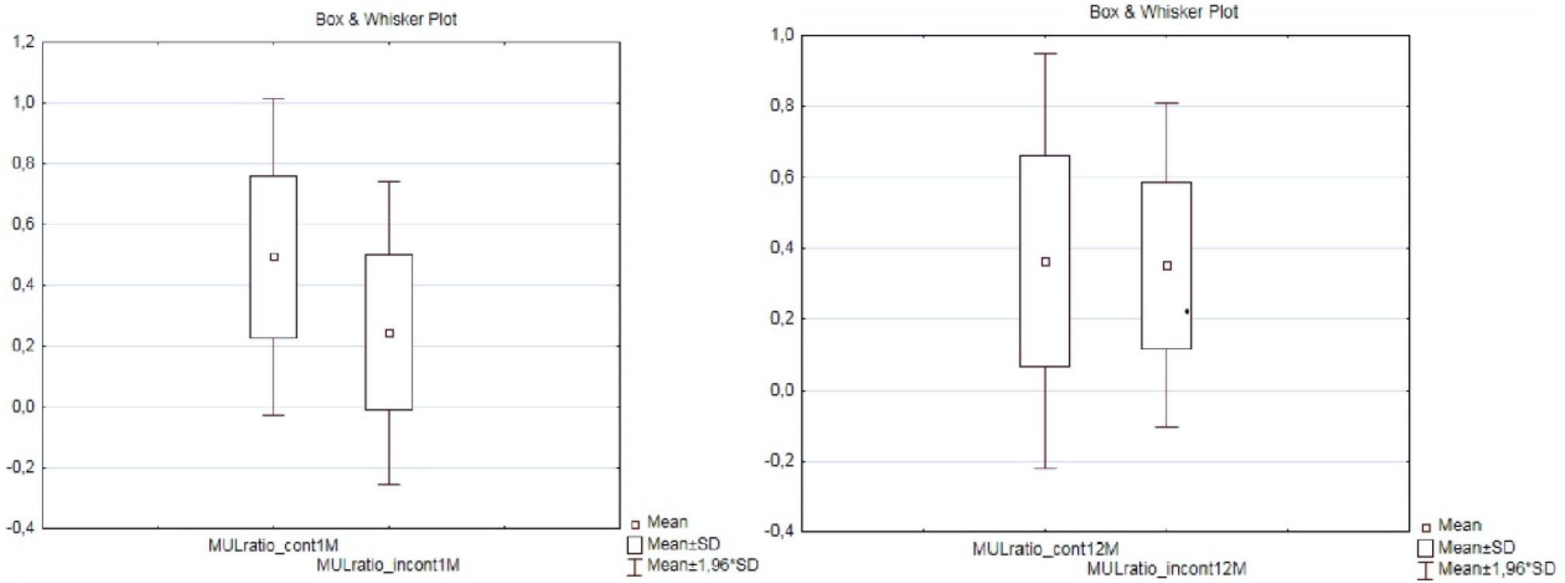
Comparison of percent change in membranous urethra length (MULratio) between subgroups of patients continent and incontinent one and twelve months post laparoscopic radical prostatectomy; (CI 95%, p = 0.00003).

**Figure 7:**
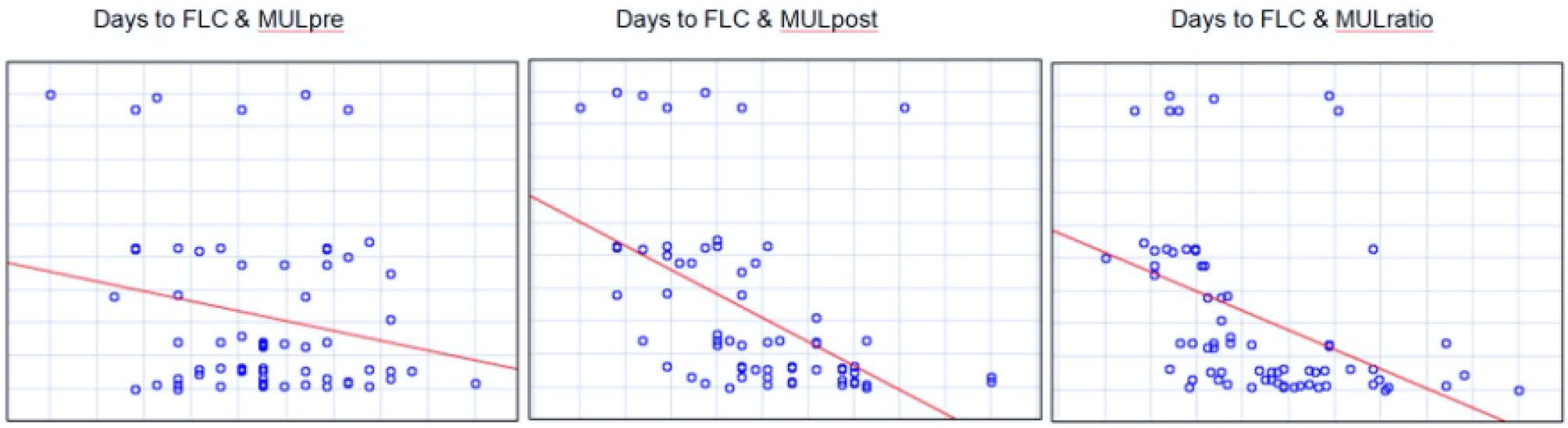
Spearman’s rank correlations test between membranous urethra length measured preoperatively (MULpre), postoperatively (MULpost) and percent change of membranous urethra length (MULratio) and time to continence recovery—days to final level of continence (Days to FLC) in the group of patients continent 12 months after laparoscopic radical prostatectomy. Strongest statistically significant inverse correlation (R—0.6, p < 0.000005) was shown between MULpost and time to continence recovery in cumulative 12 months follow-up period.

Median postoperative MUL (MULpost) was 26 mm (IQR, 20–30), range 13-46 mm and in predominant number of patients (92.8%) was longer than preoperative. MULpost was measured as a distance between the entrance of the urethra into the penile bulb to the level of clearly visible in TPUS anechocic fluid in the bladder, thus representing the entire length of posterior urethra closed at rest. Each patient had the measurement taken twice, on the day of the catheter removal and about 1–3 months after the surgery. These results were comparable and only the postoperative urethral length without the catheter was taken into the final analysis. A larger MULpost is most probably the effect of precise apical preparation and preservation of a urethral segment previously covered by prostate apex parenchyma, a suburethral suture preventing distal retraction of the urethral stump and preservation of the bladder neck. MULpost was significantly larger in a subgroup of patients that regained continence early up to one month and in a subgroup finally continent after LRP (Figs 4, 5). Higher MULratio was associated with early continence recovery but not with the final continence status (Fig. 6). Spearman’s rank test showed a strong statistically significant inverse correlation (R—0.6, 0.56; p < 0.000005) between variables MULpost, MULratio respectively and time to continence recovery in cumulative 12 months follow-up period (Fig. 7).

### Continence recovery

Overall continence recovery 1, 3, 6 and 12 months after catheter removal were 46.4%, 64.3%, 77.4% and 82.1% respectively. Patients with continence recovery within one month (n = 39, 46.4% with median 7 days) had smaller prostate and lower BMI. There was no significant difference between continent and incontinent subgroups in any time periods in terms of PSA and pathologic stage and grade. It was clearly visible that the patients who recovered the continence within one month in comparison to the cohort that regained continence later after LRP had significantly larger MULpre (average 20.8 mm versus 17.3 mm), MULpost (30.6 mm versus 20.9 mm) and higher MULratio—49% versus 24% respectively (Table 3). The longer postoperative urethral lenght may raise concerns about oncological outcome. However, positive surgical margin rate was lower in subgroup of patients continent in one month in comparison to the whole cohort 15.4% (n = 5) versus 17.8% (n = 15) respectively. Out of 15 (17.8%) patients who remained incontinent after 12 months of follow-up, 12 patients needed only one pad daily. Three other patients who needed two or more pads per day were offered additional surgical PPI management.

**Table 3:** Characteristics of patients continent and incontinent one month after laparoscopic radical prostatectomy.

	Continent at 1 month	Incontinent at 1 month
n (%)	39 (46.4)	45 (53.6)
	Mean (range)/Median (IQR)	Mean (range)/Median (IQR)
Preoperative MUL mean (mm)	20.8(14–30)/20(18.5–23)	17.3 (10–26)/16 (12–23)
Postoperative MUL mean (mm)	30.6(20–46)/30(27.5–34)	20.9 (13–39)/20(16–24)
	Mean (SD)/Median (IQR)	Mean (SD)/Median (IQR)
MUL ratio (%)	49 (± 27)/44(33–60)	24 (± 33)/18 (8.3–33)
MUL post–MUL pre (mm)	10 (± 6/10 (7–12)	3.6 (± 5)/3 (1–5)
Age mean (years)	66.1	64.4
BMI mean (kg/m^2^)	28	29
Prostate vol mean (mL)	31.5	37
PSA mean (ng/mL)	9.0	7.2
R1 n (%)	6 (15.4)	8 (17.7)

## Discussion

Many studies have evaluated preoperative MUL as an independent factor of PPI after radical prostatectomy. It has been summarized in meta-analysis^8^ which confirmed that larger preoperative MUL has a significant positive effect on overall rate of PPI and time to continence recovery and MUL measurement is recommended prior to RP. Smooth muscle sphincter as an internal layer of sphincteric complex extends from the vesical orifice to the level of perineal membrane and is coated with the striated layer from the prostatic apex caudally. Because of that anatomical fact, it seems even more important for continence recovery after RP what portion of sphincteric urethra length will remain after surgery and whether postoperative MUL could be a factor correlated with continence recovery. Most studies employed magnetic resonance imaging to measure the urethra. Due to the fact that MRI is not a standard option after surgery, data on postoperative MUL are scarce^7^. In the study of Paparel, only 72 patients from the group of 1622 after RP underwent postoperative MRI due to suspected disease progression. Although the cohort of patients analyzed was small, heterogeneous and superselected the authors concluded that both preoperative and postoperative MUL were significantly associated with time to recovery of continence. With transperineal ultrasound imaging we were able to measure the membranous urethra before and after surgery. Importanlty, the accuracy of TPUS in membranous urethra measurement and agreement with MRI outcomes was demonstrated by Mungovan et al.^15^. Two-dimensional TPUS performed with regular convex transducer is easily accessible, non-invasive, cost-effective and less time-consuming than MRI. Preoperative MUL and other predictive factors are valuable in patients counseling before surgery and with other risk factor may even have impact on patients’ decision on prostate cancer treatment plan. Such an individual risk assessment of PPI was studied by Matsushita et al.^16^ and preoperative MUL was a fulcrum of building a predictive model of urinary continence. TPUS was widely studied in stress incontinence in women^10^ but its application in PPI is limited^12,17^ and has not reached a consensus yet.

Many studies hypothesized that sparing longest possible membranous urethra can have a beneficial impact on continence recovery after LRP but were not able to measure postoperative MUL in the subststantial number of patients. To our knowledge, our study is the first one to evaluate postoperative MUL measured with TPUS as a predictive factor of continence recovery after LRP. Our analysis confirmed outcomes of many studies that larger preoperative MUL increases the rate and decrease the time to continence recovery. The subgroup of patients who regained continence early i.e. within one month had significantly larger postoperative MUL. There are many aspects during surgery that can potentially influence the chance to preserve urethra length, from localization of the tumor, anatomic variability of prostatic apex^13^, type of reconstruction of urethra support and finely experience of the surgeon. We demonstrated that in predominant cases (78 patients, 92.8%) the membranous urethra was longer after LRP than before. The percent of MUL change (MULratio) in our study proved to be a factor of early continence recovery 49% and 24% for patients continent within one month versus incontinent after one month, respectively. This finding can be useful to better manage early postoperative expectations, as many patients might need this information to accurately estimate the possibility of returning to work and full social activity. Another possible application is that postoperative MUL can be a valuable feedback for the surgeon about the quality of the apex dissection, posterior support restoration, appropriate bladder neck sparing and anastomosis. This factor can depict the efforts to preserve urethra length during surgery but it should not compromise oncological outcomes.

We consider that TPUS provides clinician with an alternative to MRI being easily accessible in an outpatient settings. In comparison to MRI, ultrasound imaging can depict pelvic structures in real time^11^. Additionally, with TPUS we were able to observe creation of funnel of an anechoic urine entering proximal urethra as a proof of an inadequate coaptation of urethral lumen during Valsalva maneuver in incontinent patients, excessive fibrosis in patients with stricture of urethra—vesical anastomosis. Furthermore, the follow up appointments with TPUS examination were used as a biofeedback visual guidance for PFMT. Physician and patient can observe voluntary contraction of pelvic floor muscles and evaluate the range of anterior movement of middle part of urethra during contraction.

### Limitations of the current study

There are several limitations of our study. First, this is a single institution and a relatively small cohort analysis. The data on the level of perioperative pelvic floor exercises was not collected, although all patients were encouraged to perform PFMT. Comorbidities were collected only generally, on the basis of American Society of Anaesthesiology (ASA) score, not considering diabetes and metabolic syndrome. Finally, continence assessment method was semiquantitative and subjective.

## Conclusion

Transperineal ultrasound appears to be a reliable tool to measure membranous urethra before and after surgery. Preoperative and postoperative MUL measured in TPUS as well as MULratio are correlated with the rate and the time to continence recovery. Furthermore, especially for clinician, MUL assessment in ultrasound could be an easily accessible method to better predict the time course of continence recovery after prostatectomy.

## Abbreviations

TPUS: Transperineal ultrasound
MUL: Membranous urethral lenght
LRP: Laparoscopic radical prostatectomy
PPI: Postprostatectomy incontinence
LUTS: Lower urinary tract symptoms
MRI: Magnetic resonance imaging
TRUS: Transrectal ultrasound
PFMT: Pelvic floor muscles training
BMI: Body mass index
ISUP: International Society of Urologic Pathologists
ASA: American Society of Anaesthesiology
EPIC: Expanded Prostate Cancer Index Composite
PSA: Prostate specific antigen
RP: Radical prostatectomy
